# Rethinking Clozapine: Lights and Shadows of a Revolutionary Drug

**DOI:** 10.3390/brainsci14010103

**Published:** 2024-01-20

**Authors:** Liliana Dell’Osso, Chiara Bonelli, Benedetta Nardi, Federico Giovannoni, Cristiana Pronestì, Ivan Mirko Cremone, Giulia Amatori, Stefano Pini, Barbara Carpita

**Affiliations:** Department of Clinical and Experimental Medicine, Section of Psychiatry, University of Pisa, 67 Via Roma, 56126 Pisa, Italy; liliana.dellosso@unipi.it (L.D.); benedetta.nardi@live.it (B.N.); f.giovannoni10@gmail.com (F.G.); cristianapronesti@gmail.com (C.P.); ivan.cremone@gmail.com (I.M.C.); g.amatori1992@gmail.com (G.A.); stefano.pini@unipi.it (S.P.); barbara.carpita1986@gmail.com (B.C.)

**Keywords:** Clozapine, treatment indications, adverse effects, regenerative effects

## Abstract

The current literature globally highlights the efficacy of Clozapine in several psychiatric disorders all over the world, with an FDA indication for reducing the risk of repeated suicidal behavior in patients with schizophrenia or schizoaffective disorder. A growing field of research is also stressing a possible broader beneficial effect of Clozapine in promoting neuroprotection and neurotrophism. However, this drug is linked to several life-threatening side effects, such as agranulocytosis, myocarditis and seizures, that limit its use in daily clinical practice. For this work, a search was performed on PubMed using the terms “Clozapine indications”, “Clozapine adverse effects”, “Clozapine regenerative effects”, and “Clozapine neuroplasticity” with the aim of reviewing the scientific literature on Clozapine’s treatment indications, adverse effects and potential regenerative role. The results confirmed the efficacy of clozapine in clinical practice, although limited by its adverse effects. It appears crucial to raise awareness among clinicians about the potential benefits of using Clozapine, as well educating medical personnel about its risks and the early identification of possible adverse effects and their management.

## 1. Introduction

Since the identification of the first antipsychotic, chlorpromazine, in 1954, the pharmaceutical industries have focused on the development of new antipsychotics in order to increase therapeutic efficacy and reduce side effects [1,2]. Up until the mid 1970s, the development of these new compounds was mainly based on their chemical structure and their ability to cause catalepsy, which was considered necessary for efficacy [2,3]. Unfortunately, the use of typical antipsychotics frequently resulted in severe and disabling movement abnormalities, such as tardive dyskinesia and dystonia, which in turn led, over the years, to the growing need for an effective antipsychotic that does not cause movement disorders [4]. 

In this framework, Clozapine was discovered, a very old but also quite modern psychotropic drug at the same time. Indeed, the discovery of Clozapine dates back to 1958, when the pharmaceutical company Wander EG synthesized three new tricyclic compounds with neuroleptic properties based on the chemical structure of Imipramine, and Clozapine was among them [5,6]. Right from the beginning, this drug showed some important differences compared to other atypical neuroleptics such as the absence of catalepsy, hyperprolactinemia and, above all, extrapyramidal side effects (EPS), such as Parkinsonian-like movement disorder and dyskinesia, after long-term use [2,4,7,8]. Unfortunately, the same absence of those previously common side effects favoured the spread of an initial scepticism towards Clozapine [2] and led many scientists to brand it as not a “true neuroleptic” [5]. For this reason, Clozapine was not initially considered as an option for the treatment of schizophrenia and was scarcely used despite clinical trials on the drug continuing both in Europe and in the U.S. [2]. During the early treatment experimental phases, side effects such as important hypotensive phenomena related to dose escalation and episodes of agranulocytosis occurring mainly in the Finnish population [1,2,8] led to the withdrawal of the drug from the market [1,2,4]. However, clinical trials continued over time and were supported by important discoveries such as the efficacy of Clozapine in children with Tourette’s Syndrome [2]. What distinguished Clozapine from other antipsychotics was its ability to promote a favourable outcome in patients with schizophrenia resistant to treatments, reducing not only positive but also negative symptoms, on which typical antipsychotics had no effect [2,4]. Indeed, one of the first randomised clinical trials compared chlorpromazine to Clozapine in a sample of 286 participants resistant to treatment with haloperidol for 6 weeks. The results showed that 30% of non-responders had a response to Clozapine, while only 4% responded to chlorpromazine [9]. In this perspective, Clozapine was approved by the Food and Drug Administration (FDA) with the indication for treatment-resistant schizophrenia under periodical haematological monitoring. The superiority of Clozapine is probably due to the fact that this compound interferes simultaneously with different and multiple receptors [4]. Although the first pharmacological studies on antipsychotics suggested the need to cover at least 70% of D2 receptors [10,11], Clozapine appears to have a coverage of less than 70% with a higher rapidity of dissociation from D2 receptors than other typical antipsychotics [4]. Dopamine receptors, which are identified in five different subtypes, are highly involved in Clozapine activity. D1 receptors, exclusively post-synaptic, particularly expressed in nigrostriatal, mesocortical and mesolimbic areas, have a moderate effect on locomotor activity. At the same time, pre- and post-synaptic D2 receptors find their place in the striatum, hippocampus, amygdala, cortical areas, hypothalamus, nucleus accumbens and olfactory areas. Pre-synaptic activation decreases locomotor activity, whereas post-synaptic D2 receptors increase it. Furthermore, these receptors control hormonal mechanisms such as prolactin and aldosterone release. Both D1 and D2 are also involved in learning and working memory, while D3, D4 and D5 seem to have less influence on these mechanisms. D3 is complementary to D2 in locomotor activity regulation, and D4 and D5 exert minimal control of movement. Clozapine seems to have a high ratio of affinity especially for D2 and D4 while, considering serotonergic receptors, it interacts with (5HT)2a receptors [12]. There is evidence in the literature that absence of EPS could be due to Clozapine’s selectivity for the mesolimbic region, where it decreases dopaminergic activity, especially influencing D1 and D2 receptors, while striatum activity remains intact [12]. Moreover, clozapine is a strong antagonist of 5HT2A receptors whose activity consists in significant reduction of dopamine release [13]. Lastly, it is also important to acknowledge the effects of Clozapine on alpha2 adrenergic, muscarinic, histaminergic receptors [8], as alpha 2 receptors play an important role in negative and cognitive symptoms [14], while the metabolite desmethylclozapine (NMDC) seems to exert action on M1 receptors that play a role in cognitive and psychotic symptoms. Moreover, both Clozapine and NMDC act on H1 and H2 receptors [8]: the stimulation of the latter proved to be very important in the treatment of resistant schizophrenia [4].

Despite the effectiveness demonstrated over many years of use, Clozapine continues to be underutilised due to its life-threatening side effects, the need for regular blood monitoring and the often-disorganised nature of patients with a need for this kind of treatment, which limits the requested compliance with the use of the drug. In this framework, our review aimed to clarify Clozapine’s possible adverse effects and their management, as well as its possible newly reported beneficial effects, not least the indications in the literature for its use in various psychiatric disorders, in order to reach a better understanding that would allow optimising Clozapine’s use in patients who could benefit from it.

## 2. Methods

From 1 September 2023 to 1 October 2023, a search was conducted on the PubMed electronic database with the following terms: Clozapine indications, Clozapine adverse effects, Clozapine regenerative effects or Clozapine neuroplasticity. For each section, we selected the related articles of the last 30 years, paying particular attention to the most recent. Considering Treatment Indications, we divided this section into 14 paragraphs concerning the main psychiatric disorders in which Clozapine is indicated, in Italy or all over the world, or off-label: Treatment resistant, Schizophrenia, Schizophrenia as first or second line, acute episodes, suicide and self injuries, Psychiatric disease in comorbidity with Parkinson, Tardive Dyskinesia, Hostility and aggression, Substance abuse disorder, psychogenic polydipsia Bipolar Disorder, Child and adolescent psychosis/Elderly/Pregnancy and Post-partum, Borderline Personality Disorder and Antisocial Personality Disorder, Obsessive–Compulsive Disorder, Autism Spectrum Disorder, Catatonia. With regard to adverse effect for each apparatus, we reported adverse effects by dividing them into very rare, rare, uncommon, common and very common. Finally, we focused on the three main regenerative aspects studied despite the fact that literacy in this field is still scarce: antiapoptotic effects, plasticity and synaptic effects, electrophysiology, other regenerative aspects.

## 3. Treatment Indications

The FDA approved Clozapine only for treatment-resistant schizophrenia, suicide prevention in schizophrenia and schizoaffective disorder. Other indications differ between different countries. All of the indications are summarized in Table 1.

### 3.1. Treatment-Resistant Schizophrenia (TRS)

Considering that the literature reports different definitions of treatment-resistant schizophrenia (TRS), in 1989, the Food and Drug Administration (FDA) in the U.S. approved the use of Clozapine for schizophrenia resistant to at least two antipsychotics, of which at least one is a first-generation antipsychotic, administered for at least six months at optimal dosage but without appreciable improvement, which is the definition we adopted in the text for TRS. Moreover, the FDA specified the need for periodic haematological controls [9,10]. Indeed, Clozapine is burdened with potentially fatal adverse effects such as agranulocytosis and myocarditis. However, it has also been shown to have a superior and peculiar role in all suicidal risk measures [20,67]. Furthermore, the recent literature still highlights the beneficial effects of Clozapine in the treatment of psychotic disorders [17,68]. Particularly, Clozapine is estimated to reduce psychometric scale scores measuring positive and negative symptoms (PANSS) by 25% in 40% of patients with schizophrenic spectrum disorders [69]. Therefore, currently, the most important international guidelines recommend the use of Clozapine only in forms of treatment-resistant schizophrenia while scrupulously performing careful haematological monitoring [16,36,70,71].

### 3.2. Clozapine as First and Second Line Treatment for Schizophrenia, Acute Episodes

The management of the first psychotic episode in schizophrenia has a strong impact on short- and long-term outcomes: while the first psychotic episode responds to a greater number of drugs, the recurrence of subsequent episodes leads to a reduced response to treatment, up to the development of real resistance [72]. In addition, any episode of relapse constitutes a neurotoxic insult [73]. Over the years, a growing number of studies, in which participants were treated with newer drugs, confirmed relapse rates of between 20 and 38%, with peaks of up to 45% when maintenance treatment was discontinued [34,74,75]. In this perspective, Clozapine, considered the most potent drug in many randomised, prospective head-to-head trials, could also be used as a first-line treatment, in order to prevent early relapses and initial therapeutic failures [18,76].

However, the off-label use of Clozapine in the early stages of psychosis remains widely discussed.

In this framework, a 2018 systematic review and metanalysis, aiming to investigate the use of this drug as a first or second line in patients without TRS or schizoaffective disorder, examined 15 papers for a total of 1114 patients: 314 treated with Clozapine and 800 receiving different antipsychotics. The authors concluded that Clozapine was superior to other antipsychotics as the first- or second-line treatment [19]. One of the studies analyzed reported a comparison between two groups of patients with schizophrenia first-episode: one received from 25 to 350 mg/day of Clozapine, while the other received 4–8 mg/day of Risperidone. Patients with Clozapine reported major adherence to the treatment, consistent improvement in positive symptoms and, at the 12th month of therapy, in negative symptoms compared to those who received Risperidone [77]. Nevertheless, since the first studies, Clozapine appeared superior to Chlorpromazine [78]. In a double-blind controlled study, patients with acute schizophrenic illness were allocated to Clozapine (300 mg/day) or Chlorpromazine for a period of 6 weeks. Clozapine showed superior efficacy in the treatment of irritability [78]. Finally, a randomized controlled trial of patients with schizophrenia compared the use of Clozapine with other atypical drugs, reporting superiority of the first in quality of life and 5 advantage points on the Positive and Negative Syndrome Scale (PANNS) [79].

### 3.3. Suicide and Self-Injuries Prevention

In the U.S., Clozapine has received the indication from the Food and Drug Administration (FDA) as the only drug for the management of suicidality in individuals with schizophrenia or schizoaffective disorder [14,15,21]. Indeed, life expectancy in patients with schizophrenic spectrum disorder is reduced by 13–20 years compared to the general population [80,81,82], and suicide is still one of the most important risk factors for mortality [25,83]. Among patients with schizophrenic spectrum disorder, 34% have mature suicidal ideation, and 5% commit suicide [84,85,86]. More generally, Clozapine revealed its superiority to other antipsychotics in preventing suicide in all psychotic disorders, including bipolar disorder with psychotic behaviours [22,23,24,83,86]. Furthermore, it has revealed its superiority also in preventing or reducing episodes of non-suicidal self-harm (NSSI) that represent a strong predictor of suicide and are very frequent in different clinical conditions such as borderline personality disorder [26,27,28]. Finally, compared with treatment continuation, discontinuation of Clozapine is associated with a significant increase in the risk of relapse, not only for psychotic manifestations, but also for suicidal symptoms [40]. A recent systematic review of over 50 selected studies confirmed the superior anti-suicide role of Clozapine in schizophrenia and schizoaffective disorder and suggested, while with limited evidence, its important role in reducing self-injuries and suicidality in patients with bipolar and borderline personality disorders [25].

### 3.4. Psychiatric Disease in Comorbidity with Parkinson’s Disease

In 2002, the European Agency for the Evaluation of Medicinal Products (EMEA) approved Clozapine for the treatment of psychotic symptoms in patients with Parkinson’s Disease (PD) after failure of standard treatments [30]. Indeed, about 25–30% of Parkinson’s patients experience psychotic symptoms, with a constant increase linked to the duration of illness and a great impact on quality of life [87]. The indication was mainly supported by data from the Parkinson Study Group of 1999 and by the FDA’s Orphan Drug Division, which demonstrated a reduction in symptoms, by 9.3 points vs. 2.6 points with placebo, with Clozapine at a mean dose of approximately 25 mg/day, as confirmed by subsequent studies [29,88,89,90,91]. It should also be considered that only one other drug, pimavanserine, has shown efficacy and tolerability in these patients [92]. Despite this, in the U.S., the FDA has not yet approved Clozapine for the treatment of psychoses in patients with PD, and the indication by the EMEA has a level B recommendation from the American Academy of Neurology (AAN) [44,93].

### 3.5. Tardive Dyskinesia

A substantial body of evidence has stressed the efficacy of Clozapine in patients with tardive dyskinesia caused by the prolonged use of other antipsychotics [31,32,34,35]. Tardive dyskinesias and late dystonias are potentially irreversible and very disabling movement disorders, characterised by repetitive and involuntary movements, present in 20–35% of patients who use antipsychotics or dopamine antagonists for at least 3 months, as delayed side effects of these drugs [94,95]. The only FDA-approved agents to treat these adverse effects are monoamine vesicular transporter type 2 (VMAT2) inhibitors [96], while another possible strategy is switching with atypical antipsychotics [95]. Among these, Clozapine may be the most effective due to its extremely low association with EPS. Study data are promising on the efficacy of Clozapine in the treatment of tardive dyskinesia induced by antipsychotics in patients with schizophrenic and non-schizophrenic spectrum disorders [31,32,33].

### 3.6. Hostility and Aggression

The Schizophrenia Patient Outcomes Research Team (PORT) guidelines recommend the use of Clozapine in patients with schizophrenic spectrum disorder with persistent hostility and aggression [36]. It is estimated that 18.5% of patients with psychotic spectrum or bipolar disorders have committed at least one physical or verbal aggression [97]. Even though 95 to 99% of violent acts committed in society do not involve psychiatric patients, violent acts contribute to increasing the stigma towards mental disorders and the people suffering from them [38], enhancing negative consequences such as legal implications and prolonged hospitalisation. Despite the scientific literature still being in its infancy about the heterogeneity of the aetiology for aggressive behaviours, generally, atypical antipsychotics have demonstrated a superior anti-aggressive effect compared to typical ones. Clozapine seems to be the most effective compound compared to drugs of the same class [37,38,39], especially within a multidimensional and behavioural pathway and in the sub-population of patients of forensic interest [40].

Even if the sedative effect of the drug could contribute to mitigate violent outbursts, it alone does not fully explain the reduction of aggressive behaviours in psychiatric patients [41,98,99]. The mechanism of action involves more than one domain, including the reduction of impulsive acts, those caused by the disease itself or by a concomitant substance abuse [39]. Clozapine has been demonstrated to be effective in reducing violent behaviours in treated patients in several randomised, double-blinded trials that objectively assessed measurable outcomes using tailored scales such as the Modified Overt Aggression Scale (MOAS) and the Barratt Impulsiveness Scale [39]. However, despite these promising data, Clozapine is still underutilised in these patients, and current APA guidelines “suggest” a low (2C) level of evidence to consider Clozapine in the treatment of aggressive behaviours in case of the absence of response to other treatments [16].

### 3.7. Substance Abuse Disorder 

Off-label uses of Clozapine include the treatment of alcohol or substance use disorder (SUD) in patients with schizophrenic spectrum disorder [36]. In 2015, The World Federation of Societies of Biological Psychiatry (WFSBP) guidelines recommended the use of Clozapine in the treatment of schizophrenia with comorbid alcohol abuse, with level B evidence and a level 3 recommendation [100]. Current studies supporting this indication are mostly non-randomized [36,44,45,101].

About 40% to 50% of patients with a schizophrenic spectrum disorder have a comorbid substance use disorder [102] and, among the substances consumed, cannabis and alcohol are the most frequent. Substance abuse is also considered responsible for 7 to 25% of psychotic onset and is also associated in the general population with the appearance of psychotic symptoms and schizophrenic spectrum disorders [52,103]. Comorbidity with SUD predisposes to worse outcomes in patients in terms of psychotic symptoms, depressive symptoms, and quality of life, as well as obviously representing a risk to patients’ health [38,104,105].

Recent Canadian [42] and Spanish [106] guidelines do not recommend the use of one specific antipsychotic in individuals with comorbid SUD, due to the lack of randomised trials. However, preliminary data suggest the usefulness of antipsychotic therapy in improving SUD, with Clozapine showing more favourable outcomes [43,101,106]. In 2023, Rafizadeh et al., in a systematic review and meta-analysis, selected 31 studies, all reporting higher odds to remain abstinent from substance abuse and decreased hospitalization when patients were treated with Clozapine [45].

### 3.8. Psychogenic Polydipsia

Clozapine has also been used in psychogenic polydipsia treatment. The only meta-analysis on the subject has failed to highlight a drug to be specifically recommended because of the scarcity of data and methodological inconsistency [107]. In this perspective, Clozapine’s role seems not to be exclusively linked to an antidopaminergic effect [46].

### 3.9. Bipolar Disorder

The quite old guidelines from the British Association for Psychopharmacology recommend Clozapine for treatment-refractory bipolar disorder (BD) [48], while more recent papers reported efficacy in symptoms ascribable to mania, depression, the intercurrence of rapid cycles and psychotic symptoms [49,50,51,108].

The efficacy of first-generation antipsychotics (FGA) in mood disorders, both with and without psychotic behaviour, is such that some authors define “atypical” antipsychotics as second-generation mood stabilisers [109].

Clozapine proved its efficacy in the treatment of acute manic episodes already 40 years ago, as well as its potentially important role as a mood stabiliser in the long-term maintenance therapy of BD [47]. However, there are currently no randomised controlled trials that have investigated the possible indications for the use of Clozapine in this context and, for this reason, it remains underused and reserved for cases of treatment-resistant BD as an off-label indication [48,49,50,51,108]. Indeed, the Canadian Network for Mood and Anxiety Treatments (CANMAT) and International Society for Bipolar Disorders (ISBD) guidelines of 2018 indicate Clozapine as a third-line drug in mania and as the only potential adjuvant in maintenance treatment [110], while an Asian consortium has accepted its use in very limited clinical scenarios [111]. However, various systematic reviews have reported that Clozapine used in monotherapy or combined with other drugs in BD is frequently associated with a reduction of both manic and depressive symptoms, extending the interval between cycles, and lowering psychotic symptoms as well as hospitalization rates and suicidal and/or aggressive behaviours, ultimately improving psychosocial functioning [50,51,53,108,112]. Moreover, Clozapine has also shown efficacy in unipolar depression, being second only to lithium in reducing the rate of re-hospitalization [54,104].

### 3.10. Psychiatric Disorders in Children, Adolescents, the Elderly, and during Pregnancy and Post-Partum

Clozapine use is not yet approved for schizophrenia treatment in children and young adults; moreover, there is a lack of studies carried out in these subpopulations due to the possible occurrence of severe adverse effects such as agranulocytosis and myocarditis [112]. However, in clinical practice, Clozapine has proven its efficacy in cases not responsive to conventional drug treatments [53]. According to a recent review, at least five randomized control trials demonstrating the effectiveness of Clozapine in refractory schizophrenia in adolescents and children are available, as well as in the mitigation of positive symptoms and reduction of aggressive behaviours and suicidal tendencies in obsessive–compulsive disorder (OCD) and, in general, of symptoms ascribable to the sphere of autistic spectrum disorder (ASD) and BD [54,55]. In this framework, the occurrence of adverse effects, although present in all studies, does not seem to differ in incidence or severity if compared to the adult population [54,56].

On the other hand, Clozapine has been reported to have positive effects in the treatment of primary psychoses and BD in the elderly. However, since elderly people potentially represent a category of greater frailty to side effects such as agranulocytosis, orthostatic hypotension, sedation, delirium and anticholinergic effects, the choice to use this compound must be well thought out and individualized, in collaboration with families and other caregivers [56].

Unfortunately, to date, data concerning the maintenance of a psychopharmacologic therapy based on Clozapine during pregnancy are even scarcer than in adolescents and the elderly. In this framework, when assessing the risk/benefit ratio of continuing a treatment with Clozapine during pregnancy and breastfeeding compared to switching to other antipsychotics, the clinician should take into account the medical history of the patient, the course of the disorder, and its overall severity, all while still bearing in mind that there are very few scientific data from studies [57,58].

### 3.11. Borderline Personality Disorder and Antisocial Personality Disorder 

Possible off-label uses of Clozapine include the treatment of personality disorders such as antisocial personality disorder (APD) and borderline personality disorder (BPD). Considering Clozapine’s effectiveness in reducing aggressive behaviours and hostility in subjects suffering from schizophrenic spectrum disorders, it has been hypothesised that it may be useful in preventing some frequent violent acts in APD [25].

BPD is a disorder that affects approximately 2.7% of adult individuals, of whom about three-quarters are female [113,114,115,116,117], and its core symptoms are often associated with various manifestations such as relational instability, anxiety, mood instability and substance abuse [113,114,115,116,117], which typically lead to severe social and functional consequences for the patient, with a reduction in quality of life. Moreover, frequently, subjects with BPD engage in repeated self-harm gestures and suicide attempts [117,118,119,120].

To date, a specific and univocal pharmacological therapy has not yet been approved for BPD, and psychotherapy comprises most of its treatment. However, recent studies have highlighted some neurobiological models underlying BPD, suggesting that some of these may benefit from treatment with psychotropic agents [117,121,122].

In clinical practice, severe BPD is often treated with more than one drug [123,124], and the most prescribed drugs in BPD are SSRIs and mood stabilizers, although for both of them, the evidence regarding their effectiveness is still scarce [117,125,126].

Among second-generation antipsychotics currently used in BPD, Clozapine could have a non-negligible clinical relevance since, as already reported, it not only has proven to be the most effective drug in the treatment of psychoses but is also widely known for its usefulness in reducing aggressive and impulsive behaviours [127] and acts of self-harm [28]. Moreover, considering the frequent occurrence of suicide attempts in the clinical history of BPD patients and the well-recognized anti-suicidal effect of Clozapine, the latter could play a crucial role in the treatment of this disorder.

Unfortunately, Clozapine has not been sufficiently investigated in BPD, and its use has been mostly described in case reports, case series and open-label studies with few cases and reviews [127,128,129,130]. For this reason, the most recent and extensive Cochrane for the pharmacological treatment of BPD decided to not include Clozapine as a therapeutic choice in BPD due to a consistent lack of literature [131].

### 3.12. Obsessive–Compulsive Disorder

The incidence of OCD in the general population does not exceed 2%, while the prevalence in schizophrenic patients reaches up to 25%, and in those with BD, it is estimated at 11% [132]. Notably, the presence of obsessive–compulsive symptoms globally worsens the prognosis of these disorders [132].

Although many authors have reported the onset or worsening of OCD during treatment with Clozapine [133,134] in recent years, many case reports have described good results with Clozapine treatment of primary OCD refractory to other treatments [59], in OCD related to intracranial surgery [59] and in OCD comorbid with BD [60].

### 3.13. Autism Spectrum Disorder 

Another off-label use of Clozapine, often hypothesised, concerns ASD. Where psychoeducational and behavioural interventions are the mainstay of ASD treatment, irritability symptoms and destructive behaviours, including non-suicidal self-injury, aggressive behaviours, tantrums and mood lability, are targets of psychopharmacological therapy [135]. Furthermore, these patients also have an increased risk of developing psychosis [136], although the exact prevalence is not known. In this framework, while alpha agonists, mood stabilizers and anticonvulsants are often used, the first line of treatment comprises second-generation antipsychotics [135]. Nonetheless, the prevalence of behavioural alterations refractory to first-line treatment with second-generation antipsychotics is high [137], and scientific evidence regarding the use of second-generation antipsychotics apart from risperidone and aripiprazole is still very limited [138].

Clozapine has been used off-label in cases of ASD resistant to other treatments with good results, especially for aggressive behaviours, as reported by a few case reports [61,62,63,139,140,141,142,143,144,145].

### 3.14. Catatonia

Catatonia is a complex neuropsychiatric syndrome, characterised by motor, affective and behavioural alterations. Even though for many years catatonia has been considered a psychiatric disorder on its way to extinction, in recent years it has benefitted from a renewed interest since it has now been discovered to occur in a wide range of diseases as a sort of ultimate common pathway for many severe mental disorders, including ASD [146,147,148,149,150,151,152]. While its exact prevalence in the general population is uncertain, in clinical settings, it is estimated to range from 3.3 to 8.9% [153,154,155] and, in acute psychiatric conditions, from 9% to 17% [156]. Catatonia appears to be a clinical condition increasingly diagnosed in ASD subjects, where its prevalence is estimated to be between 6 and 17% [157,158,159,160].

Currently, the only treatments indicated for catatonia are benzodiazepines [161] and electro convulsive therapy (ECT) [162]; however, the evidence for the effectiveness of these treatments is still limited, and often, the response is incomplete while still carrying the risks of important side effects [163].

The use of antipsychotics in the treatment of catatonia is controversial. First-generation antipsychotics are historically associated with the worsening of catatonia symptoms [164], while atypical antipsychotics have been suggested and have been used off-label in the treatment of resistant catatonia [161,165,166,167]. Among these, Clozapine proved to be the most effective [64,65,66,168], both alone and in combination with ECT [169]. Clozapine, similarly to benzodiazepines, seems to stabilise the fluctuating activity of the GABAA + B receptor, directly attacking the presumed mechanism underlying the development of catatonia [170,171,172].

### 3.15. Recommended Dosages, Administration Strategies and Monitoring Indications

Recently, some systematic reviews highlighted the broad endorsement of the use of Clozapine, particularly in the treatment of resistant schizophrenia, by as many as 17 guidelines from many countries [173,174].The recommended dosage at the start is 12.5 mg once or twice a day, on the first day, followed by daily incrementation of 25/50 mg a day, up to a dose of 250/450 mg/day, varying with consideration for several factors including gender, race, and smoking status [174]. Although the minimum effective dose is always recommended to limit adverse effects [174], it is estimated that about 60% of treatment-resistant schizophrenia patients do not respond to regular doses of the drug and maintain residual symptoms requiring dose augmentation [173]. Moreover, especially in the early stages of treatment, it is essential to monitor drug levels in the blood and possible adverse effects. For this reason, the FDA requires blood cell monitoring once a week for the first six months, then every two weeks for the next six months, and finally every month, while the EMA indicates a weekly check in the first 18 weeks and monthly thereafter. Nevertheless, the presence of an electrocardiogram at the beginning of therapy, and then once every year, is required. Finally, blood pressure and heart rate monitoring are indicated for possible transient hypotension and tachycardia phenomena [174].

## 4. Adverse Effects

Approximately 40% of patients treated with Clozapine end up discontinuing the therapy within two years due to non-adherence or the appearance of adverse events [175,176]. In this framework, the current literature usually groups adverse effects into five main categories according to their frequency: very rare (occurring in less than 0.01% of the subjects treated); rare (occurring in 0.01 to 0.1% of the subjects treated); uncommon (occurring in 0.1 to 1% of the subjects treated); common (occurring in 1 to 10% of the subjects treated); very common (occurring in more than 10% of the subjects treated). 

In clinical settings, a second classification based on the body systems involved is gaining more relevance [177]. For the sake of facilitating consultation of the different kinds of adverse effects, in this review, we will use the latter. All of the adverse effects are summarised in Table 2.

### 4.1. Cardiovascular Effects 

The most common cardiovascular adverse effect is sinus tachycardia, which results from direct effects on the sympathetic nervous system, including blockade of cardiac muscarinic M2 receptors and presynaptic adrenoceptors α2 and indirect activation of adrenoceptors β [178]. Sinus tachycardia is a rather common form of arrhythmia, with an incidence of 23.2%, whose manifestation is positively related with the dose of the compound and usually resolves in 4–6 weeks [178]. Normally, sinus tachycardia may not require any discontinuation of the therapy; however, in case of persistence, a cardio selective beta blocker (such as bisoprolol or ivabradine) may be administered. The therapy with beta blocker should be avoided in the first 6–8 weeks, for it may mask myocarditis [176].

Another Clozapine adverse effect concerning the cardiovascular system is postural hypotension. Postural hypotension generally occurs in the first 2 weeks of therapy, and most of the patients develop tolerance towards this adverse effect in 4–6 weeks. Otherwise, it may require a dose reduction, as well as hydration and low-dose fludrocortisone (starting from 100 mcg per day) [176,179]. Recent studies have reported that severe hypotension may be the cause of almost 7% of the mortality of patients in treatment [183]. On the contrary, Clozapine hypertension is quite rare and generally manifests itself after prolonged therapy. To date, its underlying mechanisms remain unclear, although it seems to be linked to a severe loss of parasympathetic activity and to a dysfunction of cardiac autonomic tone [223,224]. 

Myocarditis and cardiomyopathy represent two other life-threatening adverse effects. Myocarditis frequency ranges from 0.015% to 1.3% with 50% of cases occurring in the first weeks of therapy and an average time of onset of 2 weeks, while cardiomyopathy has a frequency between 0.1% and 0.05% extended to the entire treatment period, with a greater risk for prolonged treatments. In around 14% of the cases of myocarditis and pericarditis/pericardial effusion, patients experience eosinophilia [179,182]. The gold standard for diagnosis is biopsy. When myocarditis occurs, Clozapine treatment must be stopped. There is no global consensus for how to rechallenge a patient after myocarditis; nonetheless, the recent literature has focused on the myocarditis severity index and the speed of titration as retreatment success factors [225]. This occurrence seems to be explained by the quickness of titration that would determine an inflammatory response in the cardiac tissues. Detectable clinical features include dyspnoea (67%), fever (67%), tachycardia (58%), elevated cardiac markers (87%) and ventricular dysfunction (57%). Therefore, it is advisable to perform routine monitoring for myocarditis symptoms and signs during the first 3 months of therapy [182].

Cardiomyopathy can occur at any time, but usually occurs in prolonged treatments [176], generally after 14 months of therapy [179]. Risk factors associated with cardiomyopathy are unclear; however, it seems more frequent in patients who are taking Sodium Valproate at the time of initiation of Clozapine titration [226]. 

Lastly, Clozapine may induce an extension of the QTc interval on the ECG. In most cases, this effect has no clinical relevance and torsade de pointes are very rare; risk factors are age over 65 years and female sex [180]. Furthermore, the recent literature has also shown not only that Clozapine increases QTc, but also that plasma levels of the drug reliably predict QTc prolongation [181]. Moreover, thromboembolism is a rare but sometimes fatal complication associated with Clozapine administration that usually occurs within 6 months of starting treatment and is more common in men [184,185]. It does not appear to be dose-related [184]. Even rarer, but nevertheless documented in the literature, are myocardial infarction cases. Interestingly, the literature reported the case of a 25-year-old man without a familiar history of coronary artery disease (CAD) who had a myocardial infarction after a 8 years of treatment with 100 mg/day Clozapine for a bipolar disease diagnosis. Despite the rarity of these events, Clozapine is associated with obesity, dysregulated glucose and lipid metabolism that could induce CAD and subsequent infarction [184]. Finally, unexpected death is rare and usually concurrent with higher doses of the drug [186].

### 4.2. Central Nervous System

Among Clozapine’s adverse effects targeting the central nervous system (CNS), sedation is the most frequent, with an incidence varying from 10% to 58% and, according to few studies, sometimes reaching up to 90% of the patients treated [55,179,186,187,188]. Similarly, dizziness is common and related to its metabolite’s (norclozapine) plasma levels [189]. 

Compared to first-generation antipsychotics, Clozapine is much less likely to cause movement disorders, known as extrapyramidal side effects, which include dystonia, akathisia, parkinsonism, and tardive dyskinesia [190,191]. 

Another important central adverse effect, fatal in 5% of cases, is the onset of generalised epileptic seizures, due to the reduction of the epileptic threshold caused by the action of Clozapine [183]. To avoid Clozapine-induced seizures, suggestions in the recent literature are to start with a small doses of Clozapine and titrate slowly, monitoring its serum levels and retaining the minimal effective dose [227].

One of the most feared effects of Clozapine, shared with many other antipsychotics, is neuroleptic malignant syndrome (NMS), a rare adverse event associated with antipsychotic or other dopamine D2 receptor antagonists. The syndrome manifests with mental confusion, muscle rigidity and high fever. Most patients (92%) who had experienced neuroleptic NMS experienced the adverse event again when re-exposed to the same drug, and a high proportion of patients re-experienced it (79%) when exposed to Clozapine, even if the previous event was triggered by another antipsychotic. While being a severe syndrome and requiring tempestive treatment, it seems to have a low rate of fatal outcomes [190]. Interestingly, recent studies are suggesting a possible correlation between NMS and the co-administration of Valproic Acid [192]. Furthermore, cases of delirium and dysarthria have also been reported [193,194], sometimes related to tardive dyskinesia, with an incidence of approximately 12% [32,195]. Lastly, although controversial, the triggering role that Clozapine is sometimes reported to play in moderate-to-severe OCD should be taken into account. The increase in symptoms severity seems to be significantly associated with pre-existing OCD; a common strategy to treat Clozapine-associated OCD symptoms features the co-administration of a selective serotonin reuptake inhibitor, clomipramine or aripiprazole, often accompanied by a reduction of the Clozapine dose [181]. 

### 4.3. Haematological Side Effects

Clozapine’s effects on leukocytes are well-documented in the field; particularly, most patients experience a transient increase in neutrophil granulocytes at the start of the therapy that tends to rapidly normalise or to remain below the reference value [17]. Contrarily, Clozapine agranulocytosis typically occurs in the first 18 weeks of therapy, although cases occurring beyond 6 months have been reported. Agranulocytosis is an idiosyncratic reaction whose mechanism has not yet been elucidated; however, preliminary data attribute a key role to the association of two metabolic derivatives of Clozapine, N-desmethylclozapine and nitrenium ion, identified as triggers of granulocyte apoptosis in susceptible subjects [197]. Risk factors for Clozapine agranulocytosis are age, female sex (although not confirmed in some studies) and Asian ethnicity. It also appears that genetic polymorphisms affecting certain genes (HLA-DBQ1, HLA-B, SLCO1B3/1B7) may increase the risk. The mortality rate from Clozapine-induced agranulocytosis is estimated at 2.7–3.1%, lower than that reported for all drug-induced agranulocytosis (7–10%) [197]. The available literature reports a rate of death caused by agranulocytosis, in the last 30 years, of around 0.05% [228]. If agranulocytosis develops, Clozapine must be stopped immediately [197]. A recent systematic review selected 34 studies reporting a rechallenge of Clozapine in association with colony stimulating factor (CSF) after agranulocytosis, aiming to evaluate the efficacy and safety of this method. Results showed that agranulocytosis severity had no significant impact on the treatment outcome. However, the efficacy and safety of this strategy remain unclear [229]. 

Considering specific side effects, eosinophilia, defined as a circulating eosinophilic count greater than 500/mm^3^, occurs preferentially after 3–5 weeks of therapy and resolves spontaneously; however, it could be a symptom of serous tissue damage, such as from myocarditis [196]. Rarer are the reported cases of anaemia [198], lymphopenia [199], and thrombocytopenia [200].

### 4.4. Gastroenteric Side Effects

The most frequently reported side effect in this field is constipation, which depends on Clozapine’s anticholinergic action on intestinal motility and affects at least 75% of patients on therapy. This side effect can occur at any time and can cause serious complications, such as intestinal obstruction, faecaloma, paralytic ileus, perforation of the colon, toxic megacolon and even death [201]. Similarly, dysphagia is often a consequence of gastrointestinal hypomotility caused by Clozapine and may cause choking or pneumonia ab ingestis [202]. Another frequent gastroenteric side effect is sialorrhea—maybe due to stimulation of muscarinic receptors, blockade of alpha-2 adrenergic receptors, or inhibition of the swallowing reflex—that often fades with the progressive tolerance to therapy. Otherwise, the use of sublingual anticholinergic drugs may be taken into consideration [176,177,178,179]. Lastly, dyspepsia and gastroesophageal reflux involve approximately 20% of patients during the first 6 weeks of Clozapine therapy. Treatment with proton pump inhibitors may be indicated, but can sometimes cause a decrease in the plasma concentration of the drug [179].

### 4.5. Metabolic Side Effects

One of the most frequent metabolic effects feared by patients and clinicians is weight gain, which, according to some studies, can be found in one-third to half of the patients treated [203,204]. The increase is usually more than 7% of the patient’s body weight [230]. Fortunately, this adverse effect can be countered with an adequate diet and regular physical activity or, as a second option, the administration of controlled-release metformin may be considered [231]. Noteworthily, while most patients who experienced hyperglycaemia with Clozapine had risk factors for diabetes, hyperglycaemia was also reported in patients without risk factors. The literature includes a report on the case of a 26-year-old man with psychotic symptoms, a BMI of 26, and little abdominal fat who received Clozapine. At day 10 of titration, he reached 200 mg/day and started to experience delirium during the night, ascribed to high glucose levels in the blood, which came back within range after stopping Clozapine [232]. Indeed, the recent literature reported a diabetes diagnosis in up to 26.48% of patients treated with Clozapine and a pre-diabetic state in up to 14.63% after about 6 years of therapy [205]. Furthermore, other studies underlined a glucose intolerance incidence in about 15.4% of treated patients [206]. In most cases, discontinuation of Clozapine restored normalisation of blood glucose values. Very rarely, severe hyperglycaemia was reported to lead to ketoacidosis or hyperosmolar coma [176]. 

### 4.6. Respiratory Adverse Effects

Clozapine is associated with an increased risk of pneumonia due to some possible side effects: sialorrhea, whose presence at night favours saliva passing into the lungs, as reported by a recent case series; dysphagia, secondary to intestinal hypomotility; sedation—indeed, in order to control the drug’s sedative effect during daytime hours, a higher dose of Clozapine is often given in the evening; and immunoglobulin deficiency, which, according to some studies [233], is sometimes caused by Clozapine itself and which increases the risk of infection [207,208,233,234]. In the last two decades, accumulated evidence suggests agents derived from the benzamide group as the best solution to Clozapine-induced hypersalivation [209]. Moreover, it is known that Clozapine treatment leads to an increased risk of pulmonary embolism due to various mechanisms such as weight gain, metabolic syndrome and increased platelet aggregation. Although this event is rare, it has a high mortality rate, scored around 30% [209]. Lastly, rare cases of respiratory arrest have been reported [235]. Galappathie et al. described a case of a severe Clozapine-induced constipation leading to a silent pneumonia that caused respiratory arrest [235].

### 4.7. Systemic Side Effects

While a common systemic fever is found in about 5% of patients in the initial phase of therapy [176], prolonged alterations in temperature regulation and/or sweating and benign hyperthermia are much rarer [236]. Furthermore, cases of angioedema and leukocytoclastic vasculitis have been reported [211,212], while drug reaction with eosinophilia and systemic symptoms syndrome (DRESS) is a life-threatening hypersensitivity reaction sometimes associated with Clozapine therapy [213]. In some cases, a new administration of Clozapine triggered a new onset of symptoms, with a mortality rate about 7.4% [237].

### 4.8. Kidney Side Effects

About 20% of patients taking Clozapine may experience nocturnal enuresis; treatment consists in avoiding drinking liquids and taking caffeine before bedtime. Sometimes, it may be indicated to administer desmopressin nasal spray [176]. On the other hand, urinary retention and urinary incontinence are rare adverse effects [214], while sporadic cases of acute interstitial nephritis have been reported. Indeed, one of the most recent reports in the literature described the case of a 50-year-old schizoaffective man who developed acute interstitial nephritis induced by Clozapine, 300 mg daily, and recovered after the drug’s interruption [215]. 

### 4.9. Liver Side Effects

Although the mechanism by which Clozapine causes liver toxicity remains unknown [217], the most recognized side effect is the asymptomatic increase in hepatic transaminase aspartate aminotransferase (AST) and alanine aminotransferase (ALT), found in about 30–50% of patients taking Clozapine, which resolves in 6–12 weeks [238]. Moreover, cases of acute pancreatitis in 0.01–0.1% of the patients treated [217] and cases of fulminant hepatitis in 0.001% of the patients treated [216] have been reported. A case report highlighted the history of a 45-year-old woman with schizophrenia admitted to the hospital where she was put to a cross-titration, from Olanzapine, 2.5 mg daily, to Clozapine, 125 mg daily. After 12 days of treatment, she presented fever, an elevated white blood cell count, and elevated aspartate, alanine aminotransferase and C-reactive protein levels. She was diagnosed with Clozapine-induced hepatitis and recovered after the drug’s discontinuation. Due to the distressing reappearance of hallucinations, psychiatrists tried unsuccessfully to reintroduce Clozapine at a low dose (100 mg daily). Liver function was altered again, indicating a strong connection between the patient’s hepatitis and Clozapine [216].

### 4.10. Other Systems’ Side Effects

Recent literature in the field contains reports of a few cases of adverse effects even on other systems such as the endocrine system, where a probable relationship between Clozapine and pseudo-pheochromocytoma has been underlined through a case report [218]. Moreover, rare cases of muscle weakness, asthenia and hypotonia following Clozapine treatment have been reported [219], while it appears that, in rare cases, Clozapine could cause blurred vision and decreased visual acuity [220]. As a dermatological concern, skin reactions are reported as very rare adverse effects [221]. Lastly, a few studies also reported rare cases of priapism [210] and retrograde ejaculation [222]. Bolu et al. reported a case of a 40-year-old man who, after 3 days of low-dose Clozapine, presented retrograde ejaculation. For this reason, Clozapine was gradually decreased, and the patient shifted to another treatment [16].

## 5. Regenerative Effects

### 5.1. Anti-Apoptotic Effects

Clozapine is involved in various neuronal and cellular regenerative mechanisms. One of its most important functions is the protection from neuronal apoptosis. Clozapine’s antiapoptotic role was observed in a study where cells were exposed to ketamine, a non-competitive N-methyl-D-aspartate (NMDA-receptor) that induced cytotoxicity. Results of the study reported increasing levels of B-cell lymphoma-2 (Bcl-2), an anti-apoptotic marker, and a decrease in the pro-apoptotic cleaved form of caspase-3 in association with a decreased expression of autophagosome marker 1A/1B-light chain 3 (LC3-II), all determined by Clozapine [239]. Furthermore, since the use of ketamine has been approved as treatment for major depressive disorder (MDD), it has been revealed to cause a possible exacerbation of schizophrenic symptoms in some patients and to induce, in healthy humans, symptoms such as cognitive impairments and dissociative thoughts. However, Clozapine has been shown to reverse ketamine-induced phenomena such as alterations in glutamate metabolism and serotonin release in PFC and oxygenation levels, deficits in sensory-evoked gamma oscillations, and disruption in paired pulse inhibition [240]. Moreover, Clozapine was reported to decrease kainic acid-induced striatal lesions by 61% in mice, as a partial 5-HT(1A) agonist, confirming its role in protecting the neuronal system from excitotoxic injuries [241], and modulating key apoptosis enzymes such as Superoxide Dismutase type 1 (SOD-1) [242]. Particularly, PC12 cells use SOD-1 to protect themselves from oxygen radicals, while the p75 neurotrophic receptor (p75NTR) plays a major role in the apoptosis of neurons. It seems that Clozapine up-regulates SOD1 gene expression by more than 120% and down-regulates p75 neurotrophin receptor (p75NTR) mRNA levels by more than 65%, protecting neuronal cells from apoptosis [243]. Clozapine also seems to be involved in the intracellular trafficking mechanism for packaging cargo. In schizophrenia, the post-endocytic mechanism regulating surface receptor expression and lysosomal degradation, including G protein-coupled receptors (GPCRs), may be dysfunctional. Clozapine has been revealed as a modulator of mRNA gene expression for GPCRs, being effective in promoting better outcomes of the disease [243].

### 5.2. Plasticity and Synaptic Effects

A study from Critchlow et al. (2006) compared the effects of atypical and typical antipsychotics (in particular, haloperidol and Clozapine, in rat hippocampal cells). Results showed that the administration of 1.0 μM of Clozapine increased and enriched spinophilin protein by 70%, postsynaptic proteins such as shanK1a-puncta by 26%, and dendrite density by 59%. Furthermore, Clozapine increased filopodia. Conversely, 0.1 μM of haloperidol led to a huge decrease in all of these factors involved in neuronal synapsis, confirming the predominant role of atypical drugs such as Clozapine in determining synaptic plasticity [244]. Moreover, some studies have underline the importance of Clozapine in regulating receptor and kinase mechanisms involved in neuronal plasticity. 

Some evidence shows that the phosphorylation of glycogen synthase kinase 3 (GSK-3), which controls many neuronal functions and whose inhibition is due to the phosphorylation of the N-terminal serin mediated by protein kinase B (PKB), an upstream kinase of GSK-3, also can be mediated by some neurotransmitter receptors such as dopamine D2 and serotonin 5HT1/5HT2 receptors that are massively involved in schizophrenia and BD.

While intense stimulation of the D2 receptor leads to a dephosphorylated activation of GSK-3 via PKB, acute stimulation of 5HT1 and, conversely, blockade of 5HT2, can induce GSK-3 inhibition. Therefore, typical antipsychotics such as haloperidol, through D2 receptor antagonism, and through 5HT1 antagonism and D2 antagonistic properties, may regulate GSK3 activity via different signalling pathways [245]. Indeed, 1 μM of Clozapine is reported to increase the levels of Post-Synaptic Density 95 protein (PSD95) and the number of spines, as well as phosphorylate PKB on Thr308 and Ser473 sites, and phosphorylate GSK-3 beta Ser9, favouring neuronal regeneration and plasticity [246]. Furthermore, one of the major roles of Clozapine seems to be increasing Synapsin 1 (Syn1), neural-cell adhesion molecule 1 (NCAM1) in the hippocampus and PFC, which are massively involved in plasticity and proliferation mechanisms, and Drd2 dopamine receptors [247]. In this perspective, Brain Derived Neurotrophic factor (BDNF) regulates neuronal regeneration and synaptic plasticity. While the role of BDNF levels in depression and post traumatic stress disorder (PTSD) has been widely demonstrated, evidence of decreased BDNF in schizophrenic patients is still scarce and needs to be confirmed. In this framework, Grillo et al. (2007) observed higher BDNF levels in Clozapine-treated patients than in typical antipsychotic-treated or non-treated patients, confirming a possible role of lower BDNF levels in worsening schizophrenic symptoms [248]. 

### 5.3. Electrophysiology

A growing field of investigation has focused on possible effects that Clozapine may induce on brain plasticity trough electrophysiology, paying attention especially to the effects that it can have in medial PFC (mPFC) and hippocampus, which are known to be massively involved in schizophrenic and BD pathophysiology. Some electrophysiology studies underlined the important role of Clozapine in increasing basal dopamine levels, which, in turn, lead to a transient increase in dopamine induced by tetanic stimulation of the CA1/subicular region, mediated via D1 receptors. Indeed, mPFC receives glutamatergic projections from hippocampal the CA1/subicular region close to dopamine terminals. Therefore, dopamine could be involved in synaptic plasticity and long-term potentiation of mPFC activities such as cognition, learning and memory processes [249]. Clozapine seems to facilitate the potentiation of synaptic transmission by increasing N-methyl D-aspartic acid receptor-mediated excitatory post-synaptic currents in PFC neurons, additionally sustaining a long-term potentiation [250].

### 5.4. Other Regenerative Aspects

Clozapine also expresses its regenerative effects in different brain sites. Clozapine is involved in various neuronal and cellular regenerative mechanisms. Particularly, depending on its dose, it seems that Clozapine’s metabolites, such as desmethylclozapine, decreased viral lytic genes BZLF1, BRLF1, BMLF1 in induced Burkitt Lymphoma cells. In this perspective, Clozapine may be very useful in facilitating the comprehension of the viral lytic switch in order to develop treatments for diseases caused by Epstein Barr Virus [251].

## 6. Future Perspectives and Personalized Medicine

Despite its effectiveness, Clozapine still continues to be underutilized. In the framework of a personalized approach that is now affecting all branches of medicine, a recent systematic review selected articles documenting the attitudes, perceptions and experiences of patients who received Clozapine and their caregivers with the aim of enhancing the future use of this drug. Almost all of the selected articles reported greater patient adherence and satisfaction with Clozapine compared to treatment with common antipsychotics. Nevertheless, caregivers’ opinions are crucial. Several articles selected on this topic reported increased caregiver satisfaction with the findings of a better quality of life, level of functioning and premorbid personality regained by the patient. However, there are still many patients who decide to discontinue the treatment. Among the main causes, the authors reported the difficulty in carrying out continuous clinical monitoring, especially in the first phase of treatment, but, above all, having to deal with several side effects on which patients and caregivers do not feel adequately informed. In this perspective, modern psychiatry aims to improve the information provided by clinicians to patients and especially to caregivers, thus promoting adherence to treatment in an increasing number of patients with little insight into the disease [252].

## 7. Conclusions

The current literature globally highlights the striking efficacy of Clozapine. Indeed, over a dozen antipsychotics have been marketed in the 30 years following the first important Clozapine trial in the U.S., but none have been able to outperform Clozapine’s effectiveness [4]. Moreover, to date, Clozapine is the only drug with FDA approval for lowering the risk of repeated suicidal conduct in patients with schizophrenia or schizoaffective disorder [20], having demonstrated its ability to reduce the number of suicide attempts and hospitalizations and to improve all measures of suicidality [20].

Despite Clozapine’s effectiveness, it has several life-threatening side effects, such as agranulocytosis, myocarditis and seizures, that limit its use in daily clinical practice. Furthermore, the challenges of starting the drug safely, the need for routine blood checks, and the often-disorganised nature of people needing Clozapine have all contributed to its underuse [69]. Those issues have resulted in the use of high doses of other antipsychotics and/or polypharmacy with several antipsychotics, all of which significantly increase the risk of side effects while only partially improving clinical symptoms in treatment-resistant patients. 

In conclusion, even though many studies have highlighted how up to 40% of all individuals with schizophrenia and associated disorders could benefit from Clozapine, only a much lower percentage are actually treated with it, with an average delay of 48 months [10], ultimately resulting in a negative impact on long-term prognosis [253]. Of note, the issue of adverse effects may also have limited the development of research on the use of Clozapine for other psychiatric conditions, for which the literature remains scarce. However, the limited data have also highlighted a possible benefit of the employment of Clozapine for conditions such as BD, BPD or even ASD, thus underscoring the need to deepen investigation in the field [25,49,50,51,61,62,63,108,115,116,117,118,119,120,139,140,141,142,143,144,145]. Although preliminary, several findings suggest a broader beneficial effect of Clozapine, which may also promote neuroprotection and neurotrophism [239,240,241,242,243,244,245,246,247,248,249,250,251].

Globally, it appears crucial to raise awareness among clinicians about the benefits of using Clozapine, and on the other hand, train medical personnel in the early identification of possible adverse effects and in their management.

## Figures and Tables

**Table 1:** Treatment Indications.

Disorder/Cluster Symptoms	Indications	Off-Label Use	Latest References
Treatment-resistant schizophrenia (TRS)	Resistance to at least two antipsychotics, of which at least one is a first-generation antipsychotic, administered for at least six months at optimal dosage but without appreciable improvements		FDA, 2017 [15]; Keepers et al., 2020 [16]
Schizophrenia as first or second line, acute episodes		First psychotic episode, as first- or second-line treatment	Remington et al., 2013 [17]; Üçok et al., 2015 [18]; Okhuijsen-Pfeifer et al., 2018 [19];
Suicide and self-injuries prevention	Suicidality in individuals with schizophrenia or schizoaffective disorder		Meltzer et al., 2003 [20]; FDA, 2017 [15]; Novartis, 2019 [21];
		Suicidality in all psychotic disorders	Pompili et al., 2016 [22]; Wilkwoska et al., 2019 [23]; Forte et al., 2022 [24]; Masdrakis and Baldwin, 2023 [25];
		Episodes of NSSI in different clinical conditions	Ma et al., 2018 [26]; Jansen et al., 2021 [27]; Yang et al., 2022 [28];
Psychiatric disease in comorbidity with Parkinson’s disease	Psychotic symptoms, after failure of standard treatments		Parkinson Study Group, 1999 [29]; European Medicines Agency 2002 [30]
Tardive dyskinesia		Tardive dyskinesia caused by the prolonged use of other antipsychotics (with or without schizophrenia and related disorders)	Mentzel et al., 2018 [31]; Pardis et al., 2019 [32]; Lee et al., 2019 [33]; Rubio et al., 2020 [34]; Wong et al., 2022 [35];
Hostility and aggression	Persistent hostility and aggression in schizophrenic spectrum disorders		Buchanan et al., 2009 [36];
		Aggressive behaviours in psychiatric patients	Citrome and Volavka, 2014 [37]; Faay et al., 2018 [38]; Strassnig et al., 2019 [39]; Keepers et al., 2020 [16]
		Aggressive behaviours in forensic patients	Patchan et al., 2018 [40]
Substance abuse disorder (SUD)	Alcohol abuse in schizophrenic patients		Hasan et al., 2015 [41]
		SUD in patients with schizophrenic spectrum disorder	Arranz et al., 2018 [42]; Krause et al., 2019 [43]; Wagner et al., 2021 [44]; Rafizadeh et al., 2023 [45]
Psychogenic polydipsia		After the failure of different treatments	Fujimoto et al., 2016 [46]; Rubio et al., 2020 [34]
Bipolar disorder (BD)	Acute manic episodes, as third-line drug		Yatham et al., 2018 [47]
		Treatment-refractoriety, as maintenance therapy	Goodwin, 2009 [48]; Li et al., 2015 [49]; Delgado et al., 2020 [50]; Fornaro et al., 2020 [51]
		Depressive episodes with psychotic symptoms	Tiihonen et al., 2017 [52]
Child and adolescent psychosis/elderly/pregnancy and post-partum		Adolescents and children in similar clinical scenarios of adults, including TRS, psychotic symptoms, suicidality, aggressive behaviours, BD, OCD and ASD	Lee et al., 2018 [53]; Rachamallu et al., 2019 [54]; Adnan et al., 2022 [55]
		Elderly, same indications of adults, focusing on frailty to side effects	Rezembrink et al., 2022 [56]; Yang et al., 2022 [28]
		Pregnancy and post-partum, balancing pros and cons	Beex-Oosterhuis et al., 2021 [57]; Thanigaivel et al., 2022 [58]
Borderline personality disorder and antisocial personality disorder		Prevention of violent acts and reduction of hostility in antisocial personality disorder	Masdrakis and Baldwin, 2023 [25]
		Aggressive and impulsive behaviours in borderline personality disorder	Yang et al., 2022 [28]
		Suicidality and self-harm in borderline personality disorder	Yang et al., 2022 [28]
Obsessive–compulsive disorder (OCD)		Treatment refractoriety	Baptista et al., 2018 [59]
		OCD related to intracranial surgery	Baptista et al., 2018 [59]
		Comorbidity with BD	Poyurovsky et al., 2020 [60]
Autism spectrum disorder (ASD)		ASD resistant to other treatments, especially in patients with aggressive behaviours	Yalcin et al., 2016 [61]; Poyraz et al., 2016 [62]; Sahoo et al., 2017 [63]
Catatonia		Resistance to standard treatments	Tabbane et al., 2016 [64]; Saini et al., 2022 [65]; Caroff et al., 2022 [66]

**Table 2:** Adverse effects.

Body System	Adverse Effect	Frequency	References
Cardiovascular	Sinus tachycardia	Very common	Adeyamo 2020 Yang et al., 2022 [178]; Delgado 2020 [50]
	Postural hypotension	Common	Russo 2018 [179]; Flanagan 2020 [176]
	QTc extension	Common	Grande 2011 [180]; Kim 2022 [181]
	Myocarditis	Rare	Russo 2018 [179]; Bellissima 2020 [182]; De Leon 2020 [183]
	Cardiomyopathy	Rare	Russo 2018 [179]; Flanagan 2020 [176]
	Pericarditis	Rare	Russo 2018 [179]; De Leon 2020 [183]
	Thromboembolism	Rare	Wang 2020 [184]; Pallares Vela 2021 [185]
	Myocardial infarction	Very rare	Rose 2020 [186]
	Unexpected death	Very rare	Rose 2020 [186]
Central Nervous System	Sedation	Very common	Perdigues 2016 [187]; Sauras 2016 [188]; Russo 2018 [179]; Adnan 2022 [55];
	Dizziness	Very common	Gurcan 2021 [189];
	Generalised epileptic seizures	Common	De Leon 2020 [183];
	Movement disorders	Rare	Dean and Kane 2012 [190]; Yuen 2021 [191];
	Neuroleptic malignant syndrome	Rare	Lally 2019 [192]; Uvais 2022 [193];
	Delirium	Rare	Shvartsur 2019 [194]; Das 2021 [195]
	Dysarthria	Rare	Shvartsur 2019 [194]; Das 2021 [195];
	OCD symptoms	Rare	Kim 2020 [181];
Haematological	Transient neutrophilic granulocytosis	Common	Flanagan 2020 [176];
	Eosinophilia	Common	Demelo-Rodriguez 2019 [196]
	Agranulocytosis	Uncommon	Mijovic and MacCabe 2020 [197]
	Anaemia	Rare	Eleftheriou 2020 [198]
	Lymphopenia	Very rare	Abanmy 2014 [199]
	Thrombocytopenia	Very rare	Kate 2013 [200]
Gastroenteric	Constipation	Very common	Cohen 2012 [201]
	Sialorrhea	Very common	Russo 2018 [179]; Flannagan 2020 [176]
	Dysphagia	Rare	Cicala 2019 [202]
	Dyspepsia	Rare	Russo 2018 [179]
	Gastroesophageal reflux	Rare	Russo 2018 [179]
	Intestinal obstruction	Very rare	Cohen 2012 [201]
	Paralytic ileus	Very rare	Cohen 2012 [201]
	Toxic megacolon	Very rare	Cohen 2012 [201]
	Intestinal perforation	Very rare	Cohen 2012 [201]
Metabolic	Weight gain	Very common	Umbricht 1994 [203] Hummer 1995 [204]
	Hyperglycaemia	Rare	McGrath 2022 [205]
	Diabetes	Rare	McGrath 2022 [205]
	Glucose intolerance	Rare	Inada 2018 [206]
Respiratory	Pneumonia	Rare	Trifirò 2010 [207] Kaplan 2018 [208]
	Pulmonary embolism	Very rare	Wang 2020 [184]; Pallares Vela 2021 [185];
	Respiratory arrest	Very rare	Galappathie and Khan 2014 [209]
Systemic	Fever	Common	Flanagan 2020 [176]
	Hyperthermia	Common	Burk and Nelson 2020 [210]
	Angioedema	Very rare	Mukherjee 2019 [211]; Gurbuz 2020 [212]
	Leukocytoclastic vasculitis	Very rare	Mukherjee 2019 [211]; Gurbuz 2020 [212]
	Drug reaction with eosinophilia and systemic symptoms (DRESS)	Very rare	Sanader 2019 [213]
Kidney	Nocturnal enuresis	Common	Russo 2018 [179]
	Urinary retention	Uncommon	Winkler 2021 [214]
	Urinary incontinence	Uncommon	Winkler 2021 [214]
	Interstitial nephritis	Very rare	Vantipalli 2022 [215]
Liver	Transaminase elevation	Common	Wu 2014 [192]
	Fulminant hepatitis	Very rare	Shah 2023 [216]
	Acute pancreatitis	Very rare	Yildiz 2021 [217]
Other	Pseudo-pheochromocytoma	Rare	Lopez-Sanchez and Reyna-Villasmil 2016 [218]
	Muscle weakness and asthenia	Uncommon	Galletly 1996 [219]
	Blurred vision and decreased visual acuity	Uncommon	Borovik 2009 [220]
	Skin reactions	Very rare	Curtis et al., 2020 [221]
	Priapism	Very rare	Burk and Nelson 2021 [210]
	Retrograde ejaculation	Very rare	Bolu 2018 [222]

## Data Availability

All data generated or analysed during this study are included in this published article.
